# Extension of Murray’s law including nonlinear mechanics of a composite artery wall

**DOI:** 10.1007/s10237-014-0590-8

**Published:** 2014-05-10

**Authors:** Stefan B. Lindström, Ganarupan Satha, Anders Klarbring

**Affiliations:** Mechanics, Department of Management and Engineering, Institute of Technology, Linköping University, 58183 Linköping, Sweden

**Keywords:** Goal function, Murray’s law, Constrained mixture theory, Artery

## Abstract

A goal function approach is used to derive an extension of Murray’s law that includes effects of nonlinear mechanics of the artery wall. The artery is modeled as a thin-walled tube composed of different species of nonlinear elastic materials that deform together. These materials grow and remodel in a process that is governed by a target state defined by a homeostatic radius and a homeostatic material composition. Following Murray’s original idea, this target state is defined by a principle of minimum work. We take this work to include that of pumping and maintaining blood, as well as maintaining the materials of the artery wall. The minimization is performed under a constraint imposed by mechanical equilibrium. We derive a condition for the existence of a cost-optimal homeostatic state. We also conduct parametric studies using this novel theoretical frame to investigate how the cost-optimal radius and composition of the artery wall depend on flow rate, blood pressure, and elastin content

## Introduction

There is a long-standing hypothesis that the architecture of the vascular system is governed by the physiological principle of minimum work (Murray 1926; Taber 1998; Klarbring et al. 2003; Liu and Kassab 2007). The inferred target radius of an artery then becomes a function of the flow conditions within the blood vessel (Murray 1926). In the present work, we extend this original idea of Murray so that also the material composition and wall thickness of the artery are determined by a minimum work principle. This development ties together with the previous work (Satha et al. 2014), where we studied how local changes in volumetric blood flow or pressure, due to, for instance, disease, injury, and surgery, trigger growth and remodeling (Humphrey 2002) toward a homeostatic target state. In this paper, we develop a theory that determines this target homeostatic radius, wall thickness, and material composition, the artery wall being a composite of different constituents with nonlinear material properties (Holzapfel et al. 2000). In order to keep the theory as simple as possible, we assume the vessel to be of cylindrical shape, and we use a theory for thin-walled structures.

The blood vessel wall mainly consists of elastin, collagen, and smooth muscle (Boron and Boulpaep 2008, pp. 473–481). Thus, we model the vessel wall as a composite of multiple orthotropic, nonlinear elastic materials that deform together as the vessel stretches in the circumferential direction due to the transmural pressure, as described in the literature (Humphrey and Rajagopal 2002; Gleason and Humphrey 2004; Valentín and Humphrey 2009; Valentín et al. 2009; Satha et al. 2014). The target composition and radius are assumed to minimize the cost—that is, the power per unit length of blood vessel—required to maintain and pump the blood contained within the vessel and to maintain the materials of the vessel wall, as previously proposed (Taber 1998; Klarbring et al. 2003; Liu and Kassab 2007). The goal function of the system is then taken to be this cost function subject to the constraints imposed by the mechanical equilibrium of the vessel wall. Since the elastin content changes very slowly in the vascular system of adult individuals (Tsamis et al. 2013), the amount of elastin is essentially beyond the control of the growth and remodeling process. Therefore, we regard the amount of elastin as a parameter to the system. The goal function is then parameterized by the blood pressure, the volumetric flow rate, and the amount of elastin. These parameters, in turn, are functions of time, and their fluctuations lead to fluctuations of the target geometry and composition.

Experimental studies show that an increased blood pressure $p$ increases the thickness of the vessel wall through growth and that the vessel adapts to achieve a homeostatic state (Matsumoto and Hayashi 1996; Hu et al. 2007). These studies also show that changes in blood pressure affect the material composition of the vessel wall. Similarly, the volumetric flow rate $u$ has a strong impact on a blood vessel’s radius and composition: The radius $r$ is increased when the flow rate is increased, so that the shear stress of the fluid on the epithelial cells, that is, the interior lining of the vessel wall, is kept at a homeostatic state (Brownlee and Langille 1991). On a longer timescale, the material composition of the vessel wall also changes with increased flow rate (Kubis et al. 2001). It was suggested in an early work by Murray (1926) that the target dimensions of the blood vessel are governed by the minimization of metabolic power needed to maintain the materials of the vascular system and to overcome the hydrodynamic resistance from the vessel for a given demand of supplied blood. This minimization principle leads to Murray’s law

1 $$\begin{aligned} r \propto u^{1/3}, \end{aligned}$$

which is in fair agreement with the experimental data (Sherman 1981; Taber et al. 2001). Later, Murray’s law was modified by taking the metabolic cost of the vessel wall into account (Taber 1998), including the active behavior of smooth muscle. This latter approach relates the shear stress of the homeostatic state to the pressure, the thickness of the vessel wall, and the degree of smooth muscle metabolism. Klarbring et al. (2003) and Liu and Kassab (2007) have further developed the cost function approach by considering minimization of the cost for the vascular tree as a whole in their formulations.

To the knowledge of the authors, the fact that the artery wall is composed of several constituents with orthotropic, nonlinear properties (Holzapfel et al. 2000) has not been considered in previous studies of the cost-optimal geometry and composition of artery walls. Because the elastin content of the artery is essentially unchanging at the timescales of growth and remodeling (Tsamis et al. 2013), there is not a unique optimal target composition of the artery wall for a given set of flow parameters; the optimal state depends on the given amount of elastin, and its slow variations due to degradation. The target composition may then be coupled to the material properties of the composite artery wall.

To find the cost-optimal geometry and composition of an artery with a nonlinear mechanical behavior, it is necessary to consider a mechanical model of the artery wall in conjunction with a cost function derived from the power required to maintain the materials and blood flow of the artery. We briefly outline the mechanical model, based on constrained mixture theory (Humphrey and Rajagopal 2002; Gleason and Humphrey 2004; Valentín and Humphrey 2009; Valentín et al. 2009; Satha et al. 2014), in Sect. 2.1. This yields an equilibrium equation that relates the transmural pressure to the vessel geometry and composition of a homeostatic state. A description of the principle of cost-optimization for the artery wall follows in Sect. 2.3, and a goal function is subsequently formulated, whose minima correspond to a minimal cost of homeostatic states that satisfy the equilibrium equation (Sect. 2.4). We analyze how the cost-optimal state of the vessel varies with volumetric flow rate, pressure, and elastin content in Sect. 3.

## Theory

### Constrained mixture thin-walled tube theory

We consider a cylindrical tube composed of a mixture of $n$ materials, whose respective mechanical properties are represented by their strain energy functions $\psi ^k,k=1\;\ldots \;n$. A constrained mixture theory is used, implying that all constituents have the same deformation. This deformation, with respect to a given, fixed reference configuration, is represented by a circumferential strain $\lambda$ and a supposed constant axial strain $\delta$. For a pressure difference $p$ between the interior and exterior of the tube, integration of the standard radial equilibrium equation gives

2 $$\begin{aligned} p=\int \limits _{\rho _0}^{\rho _1}(\sigma _\varphi - \sigma _\rho )\frac{d\rho }{\rho }, \end{aligned}$$

where $\rho$ is the radial coordinate which varies between an inner radius $\rho _0$ and an outer radius $\rho _1$. For an incompressible material, the stress difference between circumferential stress $\sigma _\varphi$ and the radial stress $\sigma _\rho$ can, cf. Holzapfel and Ogden (2003), be written

3 $$\begin{aligned} \sigma _\varphi -\sigma _\rho =\sum _{k=1}^n\phi ^k \lambda \frac{\partial \psi ^k}{\partial \lambda }, \end{aligned}$$

where $\phi ^k$ denotes the volume fraction of constituent $k$. Introducing Eq. (3) into Eq. (2) and making a thin-walled tube assumption, cf. Satha et al. (2014) for details, result in

4 $$\begin{aligned} p=\frac{1}{2\pi \lambda \delta R^2}\frac{\partial }{\partial \lambda }\sum _{k=1}^n A^k\psi ^k, \end{aligned}$$

where $R$ is the radius of the, now thin-walled, reference configuration, and $A^k$ is the effective reference area obtained by multiplying the volume fraction $\phi ^k$ by the total reference cross-sectional area. The radius of a deformed, thin-walled tube is expressed as $r=\lambda R$.

Essentially following Baek et al. (2006), we take the effective areas to be represented by

5 $$\begin{aligned} A^{k}=A^{k}(0)Q^{k}(t)+\int \limits ^{t}_{0}\fancyscript{A}^{k} (\tau )q^{k}(t-\tau )d\tau ,\quad t \ge 0 \end{aligned}$$

where $A^{k}(0)$ is the original effective area of constituent $k, Q^{k}(t)$ is the fraction of constituent $k$ that was produced before time 0 and remains at time $t, \fancyscript{A}^{k}(t)\ge 0$ is the rate of production of effective area at time $t$, and $q^{k}(t)\ge 0$ is a monotonically decreasing survival function such that $q(0)=1$.

By assuming that materials created at different time instances contribute to the strain energy in proportion to the remaining area fractions, we obtain (Baek et al. 2006)

6 $$\begin{aligned} A^{k}\psi ^{k}(\lambda )&= A^{k}(0)Q^{k}(t)\varPsi ^{k} \left[ \lambda ^{k}(t,0)\right] \nonumber \\&+ \int \limits ^{t}_{0}\fancyscript{A}^{k}(\tau )q^{k}(t-\tau ) \varPsi ^{k}\left[ \lambda ^{k}(t,\tau )\right] d\tau , \end{aligned}$$

where, $\varPsi ^{k}\left[ \lambda ^{k}(t,\tau )\right]$ is the strain energy density with respect to a natural, stress-free configuration and characterizes the nonlinear, elastic behavior (Baek et al. 2006). Also, $\lambda ^{k}(t,\tau )$ is the stretch at time $t$ for materials produced at time $\tau$. Hence, (Baek et al. 2006)

7 $$\begin{aligned} \lambda ^{k}(t,\tau )=\frac{\lambda (t)}{\lambda (\tau )}G^k_\mathrm {h}. \end{aligned}$$

The ratio $\lambda (t)/\lambda (\tau )$ is the stretch developed during the time interval $[\tau ,t]$, and $G^k_\mathrm {h}$ is the homeostatic prestretch of constituent $k$, which means the material may attain prestretch at the time of production.

### Timescales and homeostatic conditions

We recognize different timescales in the process of growth and remodeling of the vascular system. The high-frequency scale is that of the heartbeat. It was shown in Satha et al. (2014) that Eq. (4) is approximately valid for average quantities if the change of $A^k$ is taken to be much slower than that of the heartbeat timescale. Moreover, we distinguish between two processes in the slow change in $A^k$. First, there is the change of homeostatic values. Secondly, there is the process of approaching these homeostatic target values when, say, a perturbation of the state occurs. The stability of the second type of process was previously investigated in Satha et al. (2014). Complementary to this, in the present paper, we study the target homeostatic state and its dependence on the imposed flow conditions. Such states are defined by a time-constant stretch $\lambda (\tau )\equiv \hat{\lambda }$ as well as a time-constant composition of materials $A^k \equiv \hat{A}^k$. There are two classes of constituents for which steady-state conditions are possible (Satha et al. 2014):

(i) Constituents that degrade, $Q^k,q^k \rightarrow 0$ as $t\rightarrow \infty$, and grow, $\fancyscript{A}^k > 0$.
(ii) Constituents that neither degrade, $Q^k = q^k = 1$, nor grow, $\fancyscript{A}^k = 0$.

The set of constituent indices belonging to class (i) and (ii) are denoted by $S_\mathrm {i}$ and $S_\mathrm {ii}$, respectively. Equations (5) and (6) result in (Satha et al. 2014)

8 $$\begin{aligned} \psi ^k(\lambda )=\left\{ \begin{array}{ll} \varPsi ^k\big (\frac{\lambda }{\hat{\lambda }}G^k_{{\mathrm {h}}}\big ), &{}\quad k \in S_\mathrm {i} \\ \varPsi ^k\big (\lambda G^k_0 \big ), &{}\quad k \in S_\mathrm {ii}. \end{array} \right. \end{aligned}$$

Here, $G^k_0 = G^k_\mathrm {h} / \lambda (0)$ is the initial prestretch of constituent $k\in S_\mathrm {ii}$ at $t=0$.

Introducing Eq. (8) into a time-averaged version of Eq. (4), and evaluating for $\lambda =\lambda (t)=\lambda (\tau )=\hat{\lambda }$ and for $A^k = A^k(t) = \hat{A}^k$, we get (Satha et al. 2014)

9 $$\begin{aligned} p=\frac{1}{2\pi \delta (R\hat{\lambda })^2}\sum _{k=1}^n \hat{A}^k\sigma _{\mathrm {h}}^{k}(\hat{\lambda }), \end{aligned}$$

where

10 $$\begin{aligned} \sigma _{\mathrm {h}}^{k}(\hat{\lambda }) = \left\{ \begin{array}{ll} G^k_\mathrm {h}d{\varPsi }^k(G^k_\mathrm {h}), &{}\quad k \in S_\mathrm {i}\\ G^k_0 \hat{\lambda } d{\varPsi }^k( G^k_0 \hat{\lambda }), &{}\quad k \in S_\mathrm {ii}\end{array} \right. \end{aligned}$$

is called the homeostatic stress. Note that the homeostatic state is associated with a constant homeostatic stress for materials with a finite turnover. Here and in the following, we use the notation $df(s)=df/ds$ and $d^nf(s)=d^nf/ds^n$.

### Principle of cost-optimization

As proposed by Murray (1926), it is assumed herein that the blood vessel growth and remodeling strive toward cost-optimization of the vascular system. This assumption has been widely used in previous modeling work (Taber 1998; Klarbring et al. 2003; Liu and Kassab 2007). In this work, we take the target homeostatic state to be governed by such an optimization rule.

We assume that the metabolic cost of the materials that constitute the vessel wall is proportional to the amount of each constituent, i.e., there are constants $\alpha ^k$ such that this cost per unit length of blood vessel in the homeostatic state can be written

11 $$\begin{aligned} \sum _k \alpha ^k \hat{A}^k, \end{aligned}$$

with the units of power per unit length. Since the homeostatic stress of smooth muscle is constant (Sect. 2.2), it is possible to represent the stress-dependent upkeep of smooth muscle (Taber 1998; Liu and Kassab 2007) by the constant $\alpha ^k$.

There is also a metabolic cost for the blood. This is again taken as proportional to the volume, i.e., it is proportional to $\pi r^2\delta$. Since $r=\hat{\lambda } R$, and since a constant axial stretch $\delta$ is considered, there is a constant $\beta$ such that the metabolic cost of the blood per unit length of the blood vessel can be written

12 $$\begin{aligned} \beta (R\hat{\lambda })^2. \end{aligned}$$

We have $\beta = \pi \delta \alpha ^{\mathrm {b}}$ where $\alpha ^{\mathrm {b}}$ is the metabolic power per unit volume of blood.

Finally, we take into account the energy per unit time consumed by the heart to maintain a certain volumetric flow rate. If we assume that the Hagen–Poiseuille equation governs the flow, the power per unit length of blood vessel required to overcome the viscous drag is (Taber 1998)

13 $$\begin{aligned} \frac{8\eta u^2}{\pi r^4}, \end{aligned}$$

where $u$ is the volumetric flow rate, and $\eta$ is the dynamic viscosity of the blood, which is assumed to be a Newtonian fluid. There is thus a constant $\gamma = 8\eta /\pi$ such that the cost is

14 $$\begin{aligned} \gamma u^2(R\hat{\lambda })^{-4}, \end{aligned}$$

per unit length of blood vessel. The total cost $P$ per unit time and length is obtained as the sum of these contributions, becoming

15 $$\begin{aligned} P(\hat{\lambda },\hat{A}^1,\hat{A}^2,\ldots )\! =\! \sum _k \alpha ^k\hat{A}^k\!+\!\beta (R\hat{\lambda })^2\!+\!\gamma u^2(R\hat{\lambda })^{-4}. \end{aligned}$$

### The optimization problem and its minima

The problem we are considering is thus to minimize the total cost $P$ under the constraint that the equilibrium condition, Eq. (9), is satisfied. This problem can be rewritten as an unconstrained optimization problem by taking an arbitrary $j\in S_\mathrm {i}$ and rewriting Eq. (9) as

16 $$\begin{aligned} \hat{A}^{j} \!=\! \frac{1}{\sigma _{\mathrm {h}}^{j}} \left[ 2\pi \delta (R \hat{\lambda })^2 p \!-\! \sum _{k=1}^{j-1}\hat{A}^k\sigma _{\mathrm {h}}^{k}(\hat{\lambda })\!-\! \sum _{k=j+1}^{n}\hat{A}^k\sigma _{\mathrm {h}}^{k}(\hat{\lambda })\right] .\nonumber \\ \end{aligned}$$

Thus, $\hat{A}^{j}=\hat{A}^{j}(\hat{\lambda },\hat{A}^1,\ldots ,\hat{A}^{j-1},\hat{A}^{j+1},\ldots ,\hat{A}^{n})$, and when substituted into the expression for $P$ in Eq. (15), we get the goal function

17 $$\begin{aligned}&f(\hat{\lambda },\hat{A}^1,\ldots ,\hat{A}^{j-1},\hat{A}^{j+1},\ldots ,\hat{A}^{n})\nonumber \\&\quad =P[\hat{\lambda },\hat{A}^1,\ldots ,\hat{A}^{j-1},\nonumber \\&\qquad \hat{A}^{j}(\hat{\lambda },\hat{A}^1,\ldots ,\hat{A}^{j-1},\hat{A}^{j+1},\ldots ,\hat{A}^{n}),\hat{A}^{j+1},\ldots ,\hat{A}^{n}].\nonumber \\ \end{aligned}$$

The target homeostatic state is now given by the unconstrained minimum of $f$, assuming that this minimum occurs for positive values of all variables.

The model is next simplified by assuming that the blood vessel wall consists of two constituents only: elastin, $k =\;$‘e’$\;\in S_{\mathrm {ii}}$, and components with a finite turnover including collagen and smooth muscle, $k =\;$‘t’$\; \in S_\mathrm {i}$. This classification incorporates the assumption that the elastin content is essentially constant over time (Tsamis et al. 2013), while other constituents have a substantially faster turnover, with a timescale of approximately 2 months (Nissen et al. 1978; Martufi and Gasser 2012). Smooth muscle is metabolically more expensive than collagen, and it is present in the vascular system to help pumping blood and to control high-frequency adaptation to changing demands of blood. The fraction of smooth muscle is then likely related to the fluctuations of the flow conditions rather than their time-averaged values. However, these dynamics are beyond the scope of this study, and we introduce the simplifying assumption that the ratio of the amount of collagen to the amount of smooth muscle is constant for any given artery.

For the two constituents, ‘e’ and ‘t,’ we can express the equilibrium equation (16) as

18 $$\begin{aligned} \hat{A}^{{\mathrm {t}}} = \frac{1}{\sigma _{\mathrm {h}}^{{\mathrm {t}}}} \left[ 2\pi \delta (R \hat{\lambda })^2 p - \hat{A}^{{\mathrm {e}}}G^{{\mathrm {e}}}_0 \hat{\lambda } d{\varPsi }^{{\mathrm {e}}}( G^{{\mathrm {e}}}_0 \hat{\lambda } ) \right] , \end{aligned}$$

where $\sigma _{\mathrm {h}}^{{\mathrm {t}}}$ is a constant homeostatic stress, and Eq. (10) was used to express $\sigma _{\mathrm {h}}^{{\mathrm {e}}}(\hat{\lambda })$. Substituting Eq. (18) into the total cost $P$ gives the goal function

19 $$\begin{aligned} f(\hat{\lambda })=P[\hat{\lambda },\hat{A}^{{\mathrm {t}}}(\hat{\lambda })]. \end{aligned}$$

This cost function, retaining only nonconstant terms, becomes

20 $$\begin{aligned} P(\hat{\lambda },\hat{A}^{{\mathrm {t}}})=\alpha ^{{\mathrm {t}}}\hat{A}^{{\mathrm {t}}}+\beta (R\hat{\lambda })^2+\gamma u^2(R\hat{\lambda })^{-4} \end{aligned}$$

and the gradient of the goal function is

21 $$\begin{aligned} \frac{d f}{d \hat{\lambda }} = \frac{\partial P}{\partial \hat{\lambda }}+\frac{\partial P}{\partial \hat{A}^{{\mathrm {t}}}}\frac{d \hat{A}^{{\mathrm {t}}}}{d\hat{\lambda }}. \end{aligned}$$

Straight-forward differentiation of Eqs. (20) and (18) yields

22 $$\begin{aligned}&\frac{\partial P}{\partial \hat{A}^{{\mathrm {t}}}} = \alpha ^{{\mathrm {t}}}\end{aligned}$$

23 $$\begin{aligned}&\frac{\partial P}{\partial \hat{\lambda }} = 2\beta R^2\hat{\lambda } - 4 \gamma u^2 R^{-4} \hat{\lambda }^{-5}\end{aligned}$$

24 $$\begin{aligned}&\frac{d \hat{A}^{{\mathrm {t}}}}{d\hat{\lambda }} = \frac{1}{\sigma _{\mathrm {h}}^{{\mathrm {t}}}} \bigg \{4\pi \delta R^2 \hat{\lambda } p- \nonumber \\&\quad \hat{A}^{{\mathrm {e}}}G^{{\mathrm {e}}}_0 \left[ d{\varPsi }^{{\mathrm {e}}}( G^{{\mathrm {e}}}_0 \hat{\lambda } ) + G^{{\mathrm {e}}}_0 \hat{\lambda } d^2{\varPsi }^{{\mathrm {e}}}( G^{{\mathrm {e}}}_0 \hat{\lambda } )\right] \bigg \}. \end{aligned}$$

The optimal target homeostatic composition of a blood vessel is found at a stationary minimum point defined by

25 $$\begin{aligned} \frac{d f}{d \hat{\lambda }} = 0, \quad \frac{d^2 f}{d \hat{\lambda }^2} > 0. \end{aligned}$$

Using that $\partial P/\partial \hat{A}^{{\mathrm {t}}} = \alpha ^{{\mathrm {t}}}$ is constant, the second derivative of $f$ is

26 $$\begin{aligned} \frac{d^2 f}{d \hat{\lambda }^2} = \frac{\partial ^2 P}{\partial \hat{\lambda }^2} + \alpha ^{{\mathrm {t}}} \frac{d^2 \hat{A}^{{\mathrm {t}}}}{d \hat{\lambda }^2}. \end{aligned}$$

We note that

27 $$\begin{aligned} \frac{\partial ^2 P}{\partial \hat{\lambda }^2} = 2\beta R^2 + 20 \gamma u^2 R^{-4} \hat{\lambda }^{-6} > 0. \end{aligned}$$

Then, $d^2 f/d\hat{\lambda }^2 > 0$ when $\alpha ^{{\mathrm {t}}} = 0$. If $\alpha ^{{\mathrm {t}}} > 0$, we must consider the sign and magnitude of $d^2 \hat{A}^{{\mathrm {t}}} / d \hat{\lambda }^2$:

28 $$\begin{aligned} \frac{d^2 \hat{A}^{{\mathrm {t}}}}{d \hat{\lambda }^2}&= \frac{1}{\sigma _{\mathrm {h}}^{{\mathrm {t}}}} \bigg \{4\pi \delta R^2 p -\hat{A}^{{\mathrm {e}}}(G^{{\mathrm {e}}}_0)^2 \left[ 2d^2{\varPsi }^{{\mathrm {e}}}( G^{{\mathrm {e}}}_0 \hat{\lambda } ) \right. \nonumber \\&\qquad \left. +\, G^{{\mathrm {e}}}_0 \hat{\lambda } d^3{\varPsi }^{{\mathrm {e}}}( G^{{\mathrm {e}}}_0 \hat{\lambda } )\right] \bigg \}. \end{aligned}$$

Whether or not this expression is positive at a stationary point can be evaluated when the material model is instantiated. This will be done in Sect. 3.1. However, qualitative insight can be gained by equivalently writing Eq. (28) as

29 $$\begin{aligned} \frac{d^2 \hat{A}^{{\mathrm {t}}}}{d \hat{\lambda }^2} = \frac{1}{\sigma _{\mathrm {h}}^{{\mathrm {t}}}} \left[ 4\pi \delta R^2 p - \hat{A}^{{\mathrm {e}}}\frac{d^2\sigma _{\mathrm {h}}^{{\mathrm {e}}}(\hat{\lambda })}{d \hat{\lambda }^2}\right] . \end{aligned}$$

Thus, in case the elastin stress $\sigma _{\mathrm {h}}^{{\mathrm {e}}}$ is proportional to $\hat{\lambda }$, so that the second term vanishes, the stationary point will always be a minimum point. On the other hand, if the elastin has a strain-stiffening behavior, then ${d^2 \hat{A}^{{\mathrm {t}}}}/{d \hat{\lambda }^2}$ may become negative. Particularly, this may be the case for small pressures.

If we assume that the metabolic cost of the vessel wall is much smaller than that of the blood, $\alpha ^{{\mathrm {t}}} \approx 0$. Then, $d f/d \hat{\lambda }= 0$ gives

30 $$\begin{aligned} 2\beta R^2\hat{\lambda } \!-\! 4 \gamma u^2 R^{-4} \hat{\lambda }^{-5} \!=\! 0 \quad \Leftrightarrow \quad r^3 \!=\! u \left( \frac{2 \gamma }{\beta } \right) ^{1/2},\qquad \end{aligned}$$

consistent with Murray’s law (Murray 1926). This result can be inserted into Eq. (18) to give a closed expression for the optimum amount of materials with finite turnover. For a finite metabolic cost of the vessel wall, $\alpha ^{{\mathrm {t}}} > 0$, the stretch at the stationary point of the goal function must be computed numerically for any nontrivial choice of strain energy function $\varPsi ^{{\mathrm {e}}}$.

## Results and discussion

The cost-optimal target geometry and composition of the vessel wall are found at the minimum stationary point of the goal function. The locus of this stationary point depends on the parameters of the goal function, including pressure $p$, volumetric flow rate $u$, elastin content $\hat{A}^{{\mathrm {e}}}$, and parameters related to the material model for elastin. These parameters vary within a population as well as with time for each individual due to, e.g., aging, changes in body mass, medical treatments, or the development of diseases. In Sect. 3.2, we perform parameter studies to quantify these variations in the optimal state. However, we first need to be explicit about the material model and its parameters.

### Parameter identification and material model

The parameters of our model are quantified using data for the radial artery (*arteria radialis*) and the common carotid artery (*arteria carotis communis*). Previous *in vivo* measurements on normotensive subjects are used, giving ensemble averages for the vessel radius $\bar{r}$, total area $\bar{A}$ of the cross section, average blood pressure $\bar{p}$, and volumetric flow rate $\bar{u}$, as compiled in Table 1. The composition, described by the fraction of elastin $\phi ^{{\mathrm {e}}}$ and the fraction of other materials $\phi ^{{\mathrm {t}}}$, is estimated using histological data from the literature, as described by Satha et al. (2014). We use histological data from Li et al. (2008) for the radial artery and from Sommer et al. (2010) for the carotid artery (Table 1).

**Table 1:** Parameters of the mechanical model for the radial and carotid artery

		Radial artery	Carotid artery
Flow conditions	$\bar{u}$	40.2 mL/min$^{\hbox {a}}$	334 mL/min$^{\hbox {f}}$
	$\bar{p}$	12 kPa$^{\hbox {b}}$	11.4 kPa$^{\hbox {g}}$
Morphology	$\bar{r}$	1.265 mm$^{\hbox {b}}$	2.465 mm$^{\hbox {g}}$
	$\bar{A}$	2.45 mm$^{\hbox {2b}}$	9.0 mm$^{\hbox {2h}}$
Composition	$\phi ^{{\mathrm {t}}}$	$0.837^{\hbox {c}}$	$0.69^{\hbox {i}}$
	$\phi ^{{\mathrm {e}}}$	$0.163^{\hbox {c}}$	$0.31^{\hbox {i}}$
Material parameters	$c_0$	88.8 kPa$^{\hbox {d}}$	74.1 kPa$^{\hbox {j}}$
	$c_1$	50.5 Pa$^{\hbox {d}}$	55.6 kPa$^{\hbox {j}}$
	$c_2$	$50.7^{\hbox {d}}$	$11.2^{\hbox {j}}$
	$G_{\mathrm {h}}^{{\mathrm {t}}}$	$1.167^{\hbox {d}}$	$1.10^{\hbox {j}}$
	$G_{\mathrm {h}}^{{\mathrm {e}}}$	$1.40^{\hbox {e}}$	$1.40^{\hbox {e}}$

$^{\hbox {a}}$Giannattasio et al. (2001)

$^{\hbox {b}}$Laurent et al. (1994)

$^{\hbox {c}}$ Estimated by Satha et al. (2014) using histology from Li et al. (2008)

$^{\hbox {d}}$ Numerical fit by Satha et al. (2014) to data from Laurent et al. (1994) and Girerd et al. (1998)

$^{\hbox {e}}$Valentín and Humphrey (2009), Valentín et al. (2009)

$^{\hbox {f}}$Likittanasombut et al. (2006)

$^{\hbox {g}}$Bussy et al. (2000)

$^{\hbox {h}}$Bussy et al. (2000) with a correction for a misrepresented unit

$^{\hbox {i}}$Sommer et al. (2010)

$^{\hbox {j}}$ Numerical fit using the method of Satha et al. (2014) with data from Bussy et al. (2000)

The stretches in the circumferential, radial and longitudinal directions are $\lambda ^k ,\;(\lambda ^k\delta )^{-1}$ and $\delta$, yielding the Cauchy–Green tensor (Holzapfel et al. 2000; Holzapfel and Ogden 2010)

31 $$\begin{aligned} \mathbf {C}^k = \left[ \begin{array}{ccc} (\lambda ^k)^2 &{}\quad 0 &{}\quad 0 \\ 0 &{}\quad (\lambda ^k\delta )^{-2} &{}\quad 0 \\ 0 &{}\quad 0 &{}\quad \delta ^2 \\ \end{array} \right] , \end{aligned}$$

with invariants $I^k_0 = \mathrm {tr}\,\mathbf {C}^k$ and $I^k_1 = (\lambda ^k)^2$. As previously described (Satha et al. 2014), the strain energy density of the elastin fraction is taken to be isotropic (Holzapfel and Ogden 2010):

32 $$\begin{aligned} \varPsi ^{{\mathrm {e}}}(I^{{\mathrm {e}}}_0) = \frac{c_0}{2}(I^{{\mathrm {e}}}_0 - 3), \end{aligned}$$

while the strain energy density of the composite of other constituents is taken to be orthotropic (Holzapfel and Ogden 2010):

33 $$\begin{aligned} \varPsi ^{{\mathrm {t}}}(I^{{\mathrm {t}}}_1) = \frac{c_1}{2 c_2} \left\{ \exp \left[ c_2 (I^{{\mathrm {t}}}_1 - 1)^2 \right] - 1 \right\} , \end{aligned}$$

where $c_1 > 0\,$Pa is a constant and $c_2 > 0$ is a nondimensional constant. Parameter identification for the radial artery was performed in a previous study (Satha et al. 2014) by least-squares fitting the two-constituent material model to experimental data (Laurent et al. 1994; Girerd et al. 1998), giving the material parameters shown in Table 1. Using Eq. (10), these parameters yield $\sigma _{\mathrm {h}}^{{\mathrm {t}}} = G_{\mathrm {h}}^{{\mathrm {t}}} d\varPsi ^{{\mathrm {t}}}(G_{\mathrm {h}}^{{\mathrm {t}}}) = 38.1$ kPa. The fitting procedure described by (Satha et al. 2014) is used herein to obtain the parameters of the carotid artery from the data of Bussy et al. (2000), with the Young’s modulus of the unloaded wall of the carotid artery estimated to 0.3 MPa, similar to the value for the brachial artery (Kinlay et al. 2001). The resulting material parameters for the carotid artery are compiled in Table 1 and give $\sigma _{\mathrm {h}}^{{\mathrm {t}}} = G_{\mathrm {h}}^{{\mathrm {t}}} d\varPsi ^{{\mathrm {t}}}(G_{\mathrm {h}}^{{\mathrm {t}}}) = 46.3$ kPa. We also choose the constant longitudinal stretch to be $\delta =1$.

The parameters, $\alpha ^{{\mathrm {t}}}, \beta$, and $\gamma$, of the goal function are obtained from the literature. Liu et al. (2012) estimate $\alpha ^{\mathrm {b}} = 51.7$ W/m$^3$ for human blood, giving $\beta = 0.16$ kW/m$^3$. With a Newtonian fluid assumption, the dynamic viscosity of human blood at 40 % hematocrit is $\eta = 3.2$ mPa$\cdot$s (Boron and Boulpaep 2008), giving $\gamma = 8.1\cdot 10^{-3}$ Js/m$^3$. The metabolic coefficient $\alpha ^{{\mathrm {t}}}$ is assumed to be dominated by smooth muscle and has an active and a passive component, with the active component proportional to the stress of that constituent (Taber 1998). We thus write

34 $$\begin{aligned} \alpha ^{{\mathrm {t}}} = \alpha ^{\mathrm {w}} + k^{\mathrm {w}} \sigma ^{{\mathrm {t}}}_{\mathrm {h}}, \end{aligned}$$

where $\alpha ^{\mathrm {w}}$ and $k^{\mathrm {w}}$ denote the passive and active metabolic constants, respectively. These constants were estimated by Taber (1998) to be $\alpha ^{\mathrm {w}} = 764$ W/m$^3$ and $k^{\mathrm {w}} = 0.00872$ s$^{-1}$ for the porcine carotid artery, giving $\alpha ^{{\mathrm {t}}} = 1.1$ kW/m$^3$ and $\alpha ^{{\mathrm {t}}} = 1.2$ kW/m$^3$ for the radial and carotid artery, respectively. We take these values for $\alpha ^{{\mathrm {t}}}$ as order of magnitude estimates for human arteries and investigate different values $\alpha ^{{\mathrm {t}}} = \{0.0, 0.1, 1.0\}$ kW/m$^3$ in the parametric studies below.

### Parametric studies

In this section, we consider the effects of the volumetric flow rate, pressure, and elastin content on the radius $r$ of the blood vessel and on the amount of constituents $\hat{A}^{{\mathrm {t}}}$ with a finite turnover. The parameter $\alpha ^{{\mathrm {t}}}$, controlling the cost of the ‘t’-type wall materials, is varied to highlight its effect on the vessel dimensions and composition. The target state for each set of parameters is found numerically by solving $df/d\hat{\lambda } = 0$ for $\hat{\lambda }$ using Eqs. (19) through (24), and then computing $\hat{A}^{{\mathrm {t}}}$ using Eq. (18).

From the point of view of growth stability, it is of great interest to assess whether the stationary points of the goal function are minima. With the prototypical values from Table 1, we have evaluated Eq. (28) for a wide range of the radius $0.1 \bar{r}<r<3 \bar{r}$ and the pressure $0.2 \bar{p}<p<3 \bar{p}$ and found that $d^2 \hat{A}^{{\mathrm {t}}}/d \hat{\lambda }^2 > 0$ within these ranges for both the radial and the carotid arteries. This means that the second derivative of the goal function with respect to $\hat{A}^{{\mathrm {t}}}$ is strictly positive, asserting that the corresponding stationary points are indeed minima. Note that this validation was conducted for one particular choice of material model. An enhanced strain-stiffening, e.g., due to an anisotropic elastin fraction, would lead to greater nonlinearity in the strain energy density which would threaten the existence of the minimum. Therefore, we cannot exclude that there exists some physiological conditions at which the minimum of the goal function is lost.

#### Volumetric flow rate

**Fig. 1:**
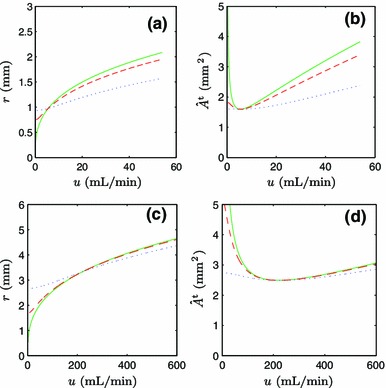
Stationary points of the goal function for different metabolic costs of the vessel wall: $\alpha ^{{\mathrm {t}}} = 0.0\,\hbox {kW/m}^3$ (*solid line*), $\alpha ^{{\mathrm {t}}} = 0.1$ kW/m$^3$ (*dashed line*), and $\alpha ^{{\mathrm {t}}}=1.0$ kW/m$^3$ (*dotted line*) in **a** the $u-r$ plane of the radial artery, **b** the $u-\hat{A}^{{\mathrm {t}}}$ plane of the radial artery, **c** the $u-r$ plane of the common carotid artery, and **d** the $u-\hat{A}^{{\mathrm {t}}}$ plane of the common carotid artery

It has been established experimentally that the volumetric flow rate has a strong impact on the blood vessel radius (Brownlee and Langille 1991; Kubis et al. 2001) and composition (Kubis et al. 2001). In our theoretical framework, this is manifested as a flow rate dependence of the stationary point of the goal function. The vessel radius $r$ and the amount of composite materials $\hat{A}^{{\mathrm {t}}}$ are plotted as functions of $u$ in Fig. 1a, b (radial artery) and Fig. 1c, d (carotid artery) for different values of $\alpha ^{{\mathrm {t}}} = \{0.0, 0.1, 1.0\}\,\hbox {kW/m}^3$ and a constant pressure $p = \bar{p}$.

When the cost of wall materials is taken to be zero, $\alpha ^{{\mathrm {t}}} = 0$, the variations of $r$ with $u$ follow Murray’s law, $r \propto u^{1/3}$ (Fig. 1a–c, solid line). Murray’s law overpredicts the average vessel radius $\bar{r}$ given in Table 1 for the average flow rate $\bar{u}$. When the wall material is assigned a finite metabolic cost, Murray’s law is modified to suppress the use of wall materials and thus reduce the radius to a more realistic value (Fig. 1a–c, dotted lines). Interestingly, this also introduces a lower bound on the vessel radius, which does not fully contract even at a vanishing flow rate.

When examining the relation $\hat{A}^{{\mathrm {t}}}(u)$ for the radial artery (Fig. 1b) and the carotid artery (Fig. 1d), it becomes clear that $\hat{A}^{{\mathrm {t}}} > 0$ for all flow rates investigated. There is a minimum of $\hat{A}^{{\mathrm {t}}}(u)$ that corresponds to a zero of $d \hat{A}^{{\mathrm {t}}}/d\hat{\lambda }$. For a constant pressure, the amount of materials in the vessel wall is a rather weak function of the flow rate.

The rise in the amount of ‘t’-material for low-volume flows corresponds to the elastin being in a state of compression, requiring additional ‘t’-material, with constant stress $\sigma _{\mathrm {h}}^{{\mathrm {t}}}$, to balance the pressure $p$. However, this may be an artifact of a too simplistic material model for elastin. The elastin forms lamellar mesostructures in the artery wall. These lamellae are likely to buckle in compression and thus dramatically reduce the strain energy stored in the elastin fraction during compression. This could significantly modify the target homeostatic states in the range of low volumetric flows.

#### Pressure

**Fig. 2:**
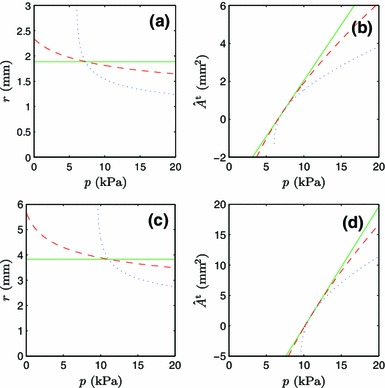
Stationary points of the goal function for different metabolic costs of the vessel wall: $\alpha ^{{\mathrm {t}}} = 0.0$ kW/m$^3$ (*solid line*), $\alpha ^{{\mathrm {t}}} = 0.1\,\hbox {kW/m}^3$ (*dashed line*), and $\alpha ^{{\mathrm {t}}}=1.0\,\hbox {kW/m}^3$ (*dotted line*) in **a** the $p-r$ plane of the radial artery, **b** the $p-\hat{A}^{{\mathrm {t}}}$ plane of the radial artery, **c** the $p-r$ plane of the common carotid artery, and **d** the $p-\hat{A}^{{\mathrm {t}}}$ plane of the common carotid artery

When the cost of the wall materials is taken to be zero, $\alpha ^{{\mathrm {t}}} = 0$, and Murray’s law governs the target state, the pressure does not have any effect on the vessel radius, as shown for both the radial and the carotid arteries by the solid lines in Fig. 2a–c. Also, it is observed in Fig. 2b–d that $\hat{A}^{{\mathrm {t}}}$ is linear in pressure, which is trivially explained by the need to balance the pressure at a constant circumferential stress $\sigma _{\mathrm {h}}^{{\mathrm {t}}}$ in the ‘t’-fraction of materials. Examining the solid lines in Fig. 2b–d, we note that $\hat{A}^{{\mathrm {t}}}$ becomes negative when the pressure is sufficiently reduced. Below this limiting pressure, no realizable homeostatic state can be found which reproduces the prediction of Murray’s law. This constitutes a lower limit of pressure for Murray’s law. This may also be an artifact of the simplistic model for the strain energy density of elastin in compression, as discussed in Sect. 3.2.1.

Under normal circumstances, with a typical pressure $p = \bar{p}$, assigning a finite cost to the wall material, $\alpha ^{{\mathrm {t}}} > 0$, leads to a more narrow blood vessel (Fig. 2a–c, dashed and dotted lines), which is closer to the measured values of $\bar{r}$ (Table 1). A narrow blood vessel reduces the force per unit length of the vessel wall and thus allows for a thinner wall, which saves expensive materials. It is interesting that the vessel radius increases when the blood pressure is reduced: A reduced blood pressure at a sustained volumetric flow rate then reduces the mechanical stability of the vessel and increases the risk of vessel collapse. The dramatic increase in the radius at low pressure is not physiological, since it occurs at states with $\hat{A}^{{\mathrm {t}}}< 0$ (Fig. 2b–d, dashed and dotted lines), which can never be achieved.

#### Elastin content

Although the elastin content is essentially constant (Tsamis et al. 2013), it may degrade over very long timescales, e.g., the lifetime of an individual. This motivates a study on how variations—particularly reductions—in elastin content affect the homeostatic target vessel geometry and composition.

**Fig. 3:**
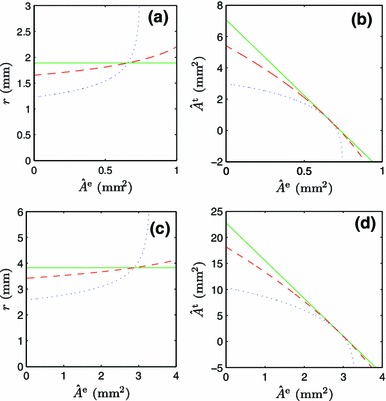
Stationary points of the goal function for different metabolic costs of the vessel wall: $\alpha ^{{\mathrm {t}}} = 0.0$ kW/m$^3$ (*solid line*), $\alpha ^{{\mathrm {t}}} = 0.1$ kW/m$^3$ (*dashed line*), and $\alpha ^{{\mathrm {t}}}=1.0$ kW/m$^3$ (*dotted line*) in **a** the $\hat{A}^{{\mathrm {e}}}-r$ plane of the radial artery, **b** the $\hat{A}^{{\mathrm {e}}}-\hat{A}^{{\mathrm {t}}}$ plane of the radial artery, **c** the $\hat{A}^{{\mathrm {e}}}-r$ plane of the common carotid artery, and **d** the $\hat{A}^{{\mathrm {e}}}-\hat{A}^{{\mathrm {t}}}$ plane of the common carotid artery

Figure 3a, c show how the radii of the radial and carotid arteries, respectively, vary with the elastin content. When $\alpha ^{{\mathrm {t}}} = 0$, the vessel radius is maintained at a constant level, owing to the fact that the elastin content does not enter into Murray’s law (Fig. 3a–c, solid line). Degradation of elastin is compensated for by an increase in the amount of other materials $\hat{A}^{{\mathrm {t}}}$. It is shown in Fig. 3b–d that $\hat{A}^{{\mathrm {t}}}$ (solid line) increases linearly when $\hat{A}^{{\mathrm {e}}}$ is reduced. That is, degraded elastin is simply replaced by other materials to balance the transmural pressure.

For the case $\alpha ^{{\mathrm {t}}} \ne 0$, elastin is replaced by metabolically more expensive materials. This is predicted to lead to a reduction of the vessel radius when elastin degrades (Fig. 3a–c, dashed and dotted lines).

### Comparison between radial and carotid artery

To demonstrate the general applicability of the proposed model, two types of arteries, the radial artery and the common carotid artery, are compared. These arteries are very different in terms of diameter and blood flow, but have a similar transmural pressure. The fraction of elastin is much greater in the carotid artery (Table 1).

The predicted variation of the vessel radius $r$ with $u$ deviates significantly from Murray’s law for the radial artery (Fig. 1a), whereas the Murray’s law appears to hold much better for the carotid artery (Fig. 1c). The same conclusions can be drawn for the amount of ‘t’-materials $\hat{A}^{{\mathrm {t}}}$ (Fig. 1b–d).

In the cases of pressure dependence and elastin content dependence, the radial and carotid arteries display the same qualitative behavior, which clearly differs from Murray’s law (Figs. 2a–d, 3a–d).

## Conclusions

The design of the vascular system is assumed to be governed by the physiological principle of minimum work (Murray 1926). It is thus an optimization process that governs the architecture of arteries. On this basis, we have formulated a theoretical frame that extends Murray’s law to include growth and remodeling, and the nonlinear mechanics of the artery wall. A goal function, novel to this application, is formulated using an expression for the power required to pump blood and the total metabolic power needed to maintain the blood and the wall of the artery.

We have shown that there exists a minimum stationary point for a wide range of the volumetric flow rate and the pressure around the prototypical parameter values for the radial and the common carotid artery. In theory, however, this minimum could be lost for a strongly strain-stiffening elastin fraction.

Taking the cost of wall materials into account reduces the radius of the target homeostatic state and also renders this target radius pressure-dependent. A reduction in the amount of elastin in the artery wall reduces the radius of the target homeostatic state.

The greatest value of the present work may be its ability to depict the variations of the target homeostatic state under dynamic flow conditions. This theoretical frame can then be integrated into models for growth and remodeling(Satha et al. 2014; Taber 1998) to capture the coupled dynamics of remodeling and fluctuation of the target state.
